# Validation of Danish registry‐cases of type 1 diabetes in women giving live birth using a clinical cohort as gold standard

**DOI:** 10.1002/edm2.374

**Published:** 2022-11-22

**Authors:** Tina Wullum Gundersen, Andreas Ebbehoj, Sine Knorr, Dorte Møller Jensen, Peter Damm, Ellen Christine Leth Løkkegaard, Elisabeth R. Mathiesen, Reimar W. Thomsen, Tine Dalsgaard Clausen

**Affiliations:** ^1^ Department of Gynecology and Obstetrics Nordsjællands Hospital and University of Copenhagen Hillerød Denmark; ^2^ Department of Endocrinology and Internal Medicine Aarhus University Hospital Aarhus Denmark; ^3^ STENO Diabetes Center Aarhus Aarhus University Hospital Aarhus Denmark; ^4^ STENO Diabetes Center Odense Odense University Hospital Odense Denmark; ^5^ Department of Gynecology and Obstetrics Odense University Hospital Odense Denmark; ^6^ Department of Clinical Research, Faculty of Health Science University of Southern Denmark Odense Denmark; ^7^ Center for Pregnant Women with Diabetes, Department of Obstetrics Rigshospitalet and University of Copenhagen Copenhagen Denmark; ^8^ Department of Clinical Medicine University of Copenhagen Copenhagen Denmark; ^9^ Center for Pregnant Women with Diabetes, Department of Endocrinology Rigshospitalet and University of Copenhagen Copenhagen Denmark; ^10^ Department of Clinical Epidemiology, Department of Clinical Medicine Aarhus University and Aarhus University Hospital Aarhus Denmark

**Keywords:** case‐identification, diabetes mellitus, pregnancy

## Abstract

**Introduction:**

The aim of this study was to validate type 1 diabetes in women giving live birth in the Danish national registries against a clinical cohort of confirmed cases (the Danish Diabetes Birth Registry [DDBR] cohort).

**Methods:**

National registries including diagnosis codes, redeemed prescriptions and background data were combined. Three main algorithms were constructed to define type 1 diabetes in women giving live birth: (1) Any diabetes diagnosis registered before delivery and before age of 30, (2) a specific type 1 diabetes diagnosis registered before delivery regardless of maternal age and (3) a ‘preexisting type 1 diabetes in pregnancy’ diagnosis registered before delivery. In additional sub‐algorithms, we added information on anti‐diabetic medicine and gestational diabetes diagnosis. We calculated positive predictive value (PPV) and completeness using the DDBR cohort as gold standard. Since DDBR included between 75 and 93% of women with confirmed type 1 diabetes giving live birth, we used quantitative bias analysis to assess the potential impact of missing data on PPV and completeness.

**Results:**

Main algorithm 2 had the highest PPV (77.4%) and shared the highest completeness (92.4%) with main algorithm 1. Information on anti‐diabetic medicine and gestational diabetes increased PPV, on expense of completeness. All algorithms varied with PPV between 65.7 and 87.6% and completeness between 73.6 and 92.4%. The quantitative bias analysis indicated that PPV was underestimated, and completeness overestimated for all algorithms. For algorithm 2, corrected PPV was between 82.1 and 94.6% and corrected completeness between 84.7 and 91.2%.

**Conclusions:**

The Danish national registries can identify type 1 diabetes in women giving live birth with a reasonably high accuracy. The registries are a valuable source for future comparative outcome studies and may also be suitable for monitoring prevalence and incidence of type 1 diabetes in women giving live birth.

## Graphical abstract

The aim of this study was to validate type 1 diabetes live births in the Danish national registries against a clinical cohort of confirmed cases.The study concludes that algorithms can reliably identify type 1 diabetes in pregnancy in health registries, with a PPV above 90% and completeness around 80%. The algorithms are well‐suited for future comparative outcome studies.

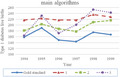

## 1 INTRODUCTION

Type 1 diabetes complicates 0.3% of pregnancies in Denmark, and the prevalence is expected to increase due to the rising incidence of childhood type 1 diabetes.^1^ Pregnancies in women with type 1 diabetes are associated with an increased risk of neonatal mortality, major congenital malformations, preeclampsia and caesarean delivery.^2^ The Danish nationwide registries have been collecting information on diagnosis, surgical treatment, demographic parameters, birth outcomes and redemption of prescription medicine on all Danish citizens since 1977. As reporting to the registries is mandatory, completeness of data is high and Danish healthcare registries provide a unique source for examining pregnancy‐outcomes among women with type 1 diabetes and their children in a population‐based setting. However, previous studies, aiming to classify diabetes in the Danish registries, points to difficulties in differentiating between type 1 and type 2 diabetes in registry data. As type 1, type 2 and gestational diabetes can be treated with the same medication and require similar attention in clinical care, it can be difficult to rely on the coding of these conditions, which is primarily due to inaccurate coding from clinicians and insufficient validation of codes before reporting to the registries. Misclassification bias, therefore, poses a fundamental challenge to epidemiological studies of live birth of mothers with type 1 diabetes, making validation studies such as the present a prerequisite to using the registries for research.^3^, ^4^, ^5^ To the best of our knowledge, the validity of data on type 1 diabetes in women giving live birth has not previously been evaluated in Danish or international registries.

Therefore, the aim of this study was to develop and validate algorithms with high accuracy for identifying type 1 diabetes in women giving live birth in registry data. The algorithms were validated against a clinical cohort of women with confirmed type 1 diabetes and live birth in Denmark (the Danish Diabetes Birth Registry [DDBR] cohort).

## 2 MATERIALS AND METHODS

### 2.1 Study design

The study was a registry‐based retrospective cohort study of the entire Danish birth cohort from 1994 to 1999. Algorithms to identify type 1 diabetes in women giving live birth were created based on diabetes codes and antidiabetic medicine and validated against a gold standard using a clinical cohort of previously confirmed cases of type 1 diabetes in women giving live birth (the DDBR cohort).

### 2.2 The DDBR cohort

During 1992 to 1999, pregnancies in Danish women with type 1 diabetes were prospectively reported to the DDBR managed by the Danish Diabetes Association. The information reported was obtained after the delivery of the child and was based on medical records. The coverage of cases from the reporting centres ranged from 75 to 93%.^6^ In this study, we used the period 1994–1999, as we wished to include the diagnosis code ‘preexisting type 1 diabetes in pregnancy’ (DO240), which was introduced with the 10th edition of the International Classification of Diseases (ICD10) in 1994.

### 2.3 Population

We used data from four nationwide Danish registries: (1) the Medical Birth Registry (1994–1999),^7^ (2) the Fertility Database (1994–1999),^8^ (3) the National Patient Registry (NPR) (1977–2010)^9^ and (4) the Registry of Medicinal Product Statistics (1995–2010).^10^ Data from the different registries were linked using the civil registration number, a unique personal identification number that all persons living in Denmark have, which is used across most public administrative and health registries. ^11^ After linkage, data were de‐identified to ensure data safety.

The Medical Birth Registry provided data on the offspring date of birth, mode of delivery and parity. The Fertility Database linked children to their parents and provided data on offspring sex, and the maternal date of birth. The NPR provided dates of maternal hospital admissions and primary and secondary ICD8 and ICD10 discharge diagnoses codes, using the Danish prefix D before the ICD‐code. NPR data were included from 1977, where the register was established. The Registry of Medicinal Product Statistics provided maternal prescriptions on redeemed insulin/insulin analogues and oral antidiabetics. The relevant codes and changes over time for diagnoses and medicine are listed in Table 1.

**TABLE 1:** Data displayed are (1) main and sub‐algorithms used to classify type 1 diabetes in women giving live birth using data from Danish registries, (2) international classification of disease (ICD) diagnosis codes and (3) changes over time for diagnoses and procedures

1) Main algorithms used to define type 1 diabetes in women giving live birth	2) ICD‐system and ATC‐codes
Algorithm 1: any diabetes diagnosis registered in NPR before delivery in the index pregnancy and registered before maternal age of 30	ICD8: 249, 250 ICD10: DE10, DE11, DE12, DE13, DE14, DP702, DH360
Algorithm 2: a specific ‘type 1 diabetes’ diagnosis registered in NPR before delivery regardless of maternal age in the index pregnancy	ICD8: 249 ICD10: DE10, DP702
Algorithm 3: a ‘preexisting type 1 diabetes in pregnancy’ diagnosis registered in NPR before delivery regardless of maternal age in the index pregnancy	ICD10: DO240
Sub‐algorithms for each algorithm	
a: Women who ever redeemed at least one prescription of insulin	Insulin (ATC‐code A10A)
b: Women who redeemed a prescription of insulin before oral antidiabetics	Insulin (ATC‐code A10A) before oral antidiabetics (ATC‐code A10B)
c: Women who ever redeemed a prescription of insulin and never redeemed a prescription of oral antidiabetics	Insulin (ATC‐code A10A) and no oral antidiabetics (ATC‐code A10B)
d: Women who never redeemed a prescription of oral antidiabetics	Oral antidiabetics (ATC‐code A10B)
e: Women who were never given a diagnosis of gestational diabetes in the index pregnancy	Gestational diabetes diagnosis given before delivery (ICD10: DO244)
f: Women who never redeemed a prescription of oral antidiabetics and who were never given a diagnosis of gestational diabetes in the index pregnancy	Gestational diabetes diagnosis given before delivery (ICD10: DO244) and no oral antidiabetics (ATC‐code A10B)

Abbreviations: ATC, anatomical therapeutic chemical; ICD, International Classification of Disease.

*Note*: ICD 8 diagnosis codes are collected from the Danish National Patient Registry from 1977 to 1993. ICD 10 diagnosis codes are collected from the Danish National Patient Registry from 1994 to 2010. ATC‐codes are collected from the Registry of Medicinal Product Statistics from 1995 to 2010.

The Danish Data Protection Agency approved the study (journal number NOH‐2016‐004). The patient consent for this study was not needed in accordance with Danish regulations.

### 2.4 Algorithms to identify type 1 diabetes in women giving live birth in the Danish registries

Based on our clinical knowledge and using registry data, we defined three main algorithms with data from the NPR in order to identify cases of type 1 diabetes in women giving live birth (Table 1):

- Algorithm 1: any diabetes diagnosis registered in NPR before delivery in the index pregnancy and registered before maternal age of 30,
- Algorithm 2: a specific ‘type 1 diabetes’ diagnosis registered in NPR before delivery regardless of maternal age in the index pregnancy,
- Algorithm 3: a ‘preexisting type 1 diabetes in pregnancy’ diagnosis registered in NPR before delivery regardless of maternal age in the index pregnancy.

In order to increase the validity of the algorithms, we applied six additional criteria based on issued medication registered in the Registry of Medicinal Product Statistics and a registered diagnosis of gestational diabetes in the index pregnancy from the NPR to each main algorithm, thereby creating 6 additional sub‐algorithms including only:

- a. Women who ever redeemed at least one prescription of insulin,
- b. Women who redeemed a prescription of insulin before a prescription of oral antidiabetics,
- c. Women who ever redeemed a prescription of insulin and never redeemed a prescription of oral antidiabetics,
- d. Women who never redeemed a prescription of oral antidiabetics,
- e. Women who were never given a diagnosis of gestational diabetes in the index pregnancy,
- f. Women who never redeemed a prescription of oral antidiabetics and who were never given a diagnosis of gestational diabetes in the index pregnancy.

In total, this resulted in 21 algorithms: 3 main algorithms and 18 sub‐algorithms (Table 1).

### 2.5 Statistics

Validation metrics included positive predictive value (PPV) and completeness where the DDBR cohort was used as the gold standard. The PPV was calculated as a proportion with the numerator containing the number of all cases identified by the algorithm (algorithm positive) who were also found in the DDBR cohort (true positive) and the denominator containing the number of all algorithm positive. Completeness (i.e., sensitivity) was defined as a proportion with the numerator containing the true positive and the denominator containing the total number of all cases in the DDBR cohort (DDBR positive).

Since DDBR only included between 75 and 93% of confirmed cases of type 1 diabetes in women giving live birth in Denmark due to resource restraints and incomplete reporting at the local centres,^6^ the gold standard we compared our algorithms with, was not complete. Therefore, we performed a standard quantitative bias analysis to estimate the impact of the missing cases. We repeated calculations for PPV and completeness for the algorithms in a quantitative bias analysis, where we corrected for the 7%–25% cases of type 1 diabetes in women giving live birth, who were missing from the DDBR cohort. We calculated a best estimate of the true PPV and completeness after correction as well as a range, wherein the true values are to be found. The potential impact of the missing cases as well as the results of the calculations are explained and presented in Appendix S1.

## 3 RESULTS

### 3.1 Population metrics

We included 310,583 mothers who had 407,157 live births during the validation‐period of 1994–1999 with an average of 67,892 deliveries per year. In the DDBR2 cohort, there were 984 confirmed live births of 796 mothers with type 1 diabetes corresponding to 0.24% of the birth cohort from 1994 to 1999.

### 3.2 The algorithms

For the main algorithms, the highest PPV of 77.4% was found among women with a specific type 1 diabetes diagnosis registered before delivery regardless of maternal age (main algorithm 2). Any diabetes diagnosis registered before delivery in the index pregnancy and before age of 30 (main algorithm 1) and main algorithm 2 shared the highest completeness of 92.4% (Table 2). The PPV and completeness of the sub‐algorithms varied between 67.8%–87.6% and 73.6%–92.4%, respectively, depending on the inclusion of additional information on issued prescriptions of insulin or oral antidiabetics and exclusion of women who had a registered gestational diabetes mellitus diagnosis code during the index pregnancy. Main algorithm 3 had the lowest PPV (65.7%) and sub‐algorithm 3.f (main algorithm 3 including only women who never redeemed a prescription of oral antidiabetics and who were never given a diagnosis of gestational diabetes in the index pregnancy) had the lowest completeness (73.6%). In general, PPV increased, and completeness decreased as extra criteria were added to the sub‐algorithms, but no criteria impacted equally across the different algorithms.

**TABLE 2:** Positive predictive value (PPV) and completeness of the main algorithms and sub‐algorithms compared with the DDBR cohort

	PPV, %	Completeness, %	TP, *n*	FP, *n*	FN, *n*	TN, *n*
Algorithm 1: any diabetes diagnosis registered in NPR before delivery in the index pregnancy and registered before maternal age of 30	67.9	92.4	909	429	75	405,744
redeemed at least one prescription of insulin	76.7	92.4	909	276	75	405,897
b redeemed a prescription of insulin before oral antidiabetics	78.7	91.2	897	243	87	405,930
c redeemed a prescription of insulin and never redeemed a prescription of oral antidiabetics	81.8	84.6	832	185	152	405,988
d never redeemed a prescription of oral antidiabetics	67.8	92.4	909	432	75	405,741
e never given a diagnosis of GDM in the index pregnancy	70.0	88.5	871	374	113	405,799
f never redeemed a prescription of oral antidiabetics and who were never given a diagnosis of GDM in the index pregnancy	73.4	83.9	826	300	158	405,873
Algorithm 2: a specific ‘type 1 diabetes’ diagnosis registered in NPR before delivery regardless of maternal age in the index pregnancy	77.4	92.4	909	266	75	405,907
redeemed at least one prescription of insulin	79.0	92.4	909	242	75	405,931
b redeemed a prescription of insulin before oral antidiabetics	75.1	91.5	900	298	84	405,875
c redeemed a prescription of insulin and never redeemed a prescription of oral antidiabetics	87.6	84.3	830	118	154	406,055
d never redeemed a prescription of oral antidiabetics	79.9	84.3	830	209	154	405,964
e never given a diagnosis of GDM in the index pregnancy	80.4	87.2	858	209	126	405,964
f never redeemed a prescription of oral antidiabetics and who were never given a diagnosis of GDM in the index pregnancy	81.6	80.0	787	177	197	405,996
Algorithm 3: a ‘preexisting type 1 diabetes in pregnancy’ diagnosis registered in NPR before delivery regardless of maternal age in the index pregnancy	65.7	84.5	831	434	153	405,739
redeemed at least one prescription of insulin	71.6	84.5	831	330	153	405,843
b redeemed a prescription of insulin before oral antidiabetics	74.3	83.3	820	284	164	405,889
c redeemed a prescription of insulin and never redeemed a prescription of oral antidiabetics	79.7	76.3	751	191	233	405,982
d never redeemed a prescription of oral antidiabetics	74.4	76.3	751	258	233	405,915
e never given a diagnosis of GDM in the index pregnancy	73.9	81.1	798	282	186	405,891
f never redeemed a prescription of oral antidiabetics and who were never given a diagnosis of GDM in the index pregnancy	79.3	73.6	724	189	260	405,984

*Note*: PPV defined as TP divided by TP + FP. Completeness defined as TP divided by TP + FN. Please note that the original DDBR cohort is missing some cases of type 1 diabetes in women giving live birth. These would have been included in the false positive or true negative columns (Appendix S1).

Abbreviations: GDM, gestational diabetes mellitus; FN, false negative; FP, false positive; PPV, positive predictive value; TN, true negative; TP, true positive.

**FIGURE 1:**
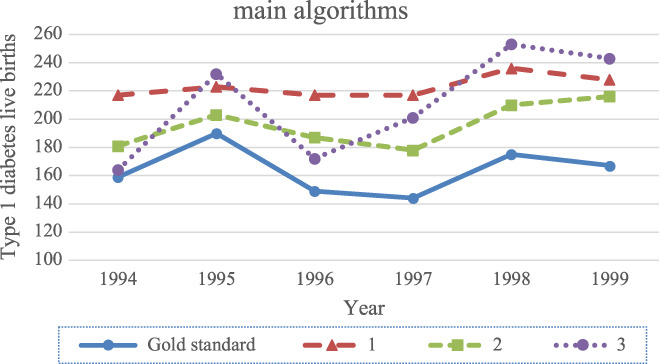
Graphic presentation of the number of cases of type 1 diabetes in women giving live birth found by main algorithm per year as well as the confirmed cases from the DDBR cohort used as gold standard. Main algorithms (1) any diabetes diagnosis registered in NPR before delivery in the index pregnancy and registered before maternal age of 30, (2) a specific ‘type 1 diabetes’ diagnosis registered in NPR before delivery regardless of maternal age in the index pregnancy, (3) a ‘preexisting type 1 diabetes in pregnancy’ diagnosis registered in NPR before delivery regardless of maternal age in the index pregnancy.

**FIGURE 2:**
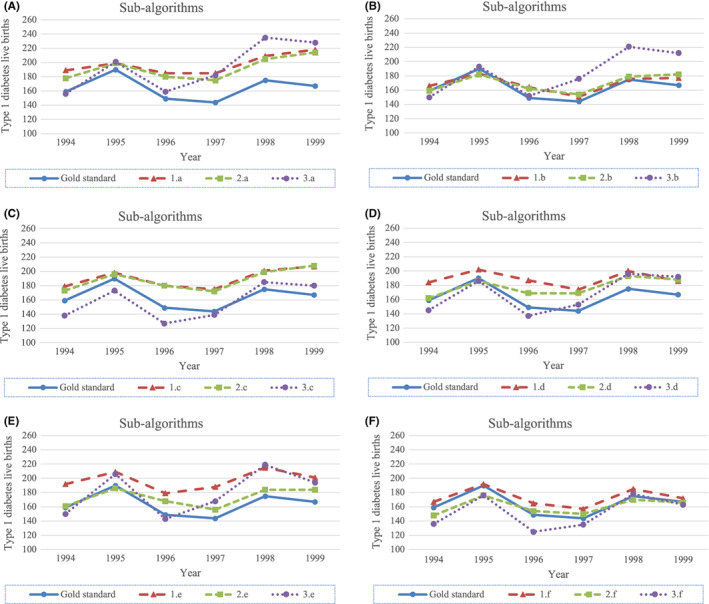
Graphic presentation of the number of cases of type 1 diabetes in women giving live birth found by main algorithm per year as well as the confirmed cases from the DDBR cohort used as gold standard. (A) women who ever redeemed at least one prescription of insulin, (B) women who redeemed a prescription of insulin before oral antidiabetics, (C) women who ever redeemed a prescription of insulin and never redeemed a prescription of oral antidiabetics, (D) women who never redeemed a prescription of oral antidiabetics, (E) women who were never given a diagnosis of gestational diabetes in the index pregnancy, (F) women who never redeemed a prescription of oral antidiabetics and who were never given a diagnosis of gestational diabetes in the index pregnancy.

Figures 1 and 2 illustrate how the main algorithms and sub‐algorithms identified varying numbers of cases, as well as how the number of identified cases fluctuated over time. The number of algorithm positive and DDBR positive cases increased in both total number and percentage of total deliveries in Denmark between 1994 and 1999 (Figure 1). Based on the DDBR cohort, cases of type 1 diabetes in women giving live birth increased 5% from 1994 to 1999 (from 159 to 167 women). Based on the main algorithms, the number increased from 1994 to 1999 with 48% (algorithm 1; from 164 to 243), 19% (algorithm 2; from 181 to 216) and 5% (algorithm 3; from 217 to 228). The smallest year‐to‐year intra‐algorithm variance was observed for sub‐algorithms based on algorithm 2 and the largest year‐to‐year intra‐algorithm variance for sub‐algorithms based on algorithm 3 (figure 2).

### 3.3 Quantitative bias analysis

As the coverage of the DDBR cohort ranged from 75 to 93%, we assume that 7%–25% of true cases of type 1 diabetes in women giving live birth are missing from the DDBR cohort. This corresponds to 74–328 missing cases of type 1 diabetes in women giving live birth in the time period 1994–1999 (Appendix S1 and Table S1). When calculating corrected estimates in the quantitative bias analysis (Appendix S1 and Figure S1), we found that PPV was generally underestimated for all algorithms and completeness was generally overestimated.^23^ Following correction for the missing cases, the best estimates on main algorithm 1 found a corrected PPV of 72.1%–86.5% (uncorrected 67.9%) and a corrected completeness of 88.3%–91.2% (uncorrected 92.4%). Best estimates for main algorithm 2 showed the highest corrected PPV of 82.1%–94.6% (uncorrected 77.4%) and shared the highest corrected completeness of 84.7%–91.2% (uncorrected 92.4%) with main algorithm 1. Best estimates for algorithm 3 had the lowest corrected PPV of 70.1%–85.4% (uncorrected 65.7%) and the lowest corrected completeness of 82.3%–83.8% (uncorrected 84.5%). The corrected PPV and completeness of the sub‐algorithms varied between 72.0%–97.0% and 66.2%–91.2%, respectively (Appendix S1 and Table S2).

## 4 DISCUSSION

In this study, we used algorithms and Danish national health registries to identify type 1 diabetes in women giving live birth among all 407,157 live births in Denmark from 1994 to 1999. All algorithms were validated against a clinical cohort of women with confirmed type 1 diabetes and live birth in Denmark as a gold standard. Algorithms based on any diabetes diagnosis code given before delivery and registered before maternal age of 30 (algorithm 1) or a specific type 1 diabetes diagnosis code regardless of maternal age (algorithm 2) had acceptable PPV and completeness, whereas algorithms based on a specific ‘preexisting type 1 diabetes in pregnancy’ diagnosis code (algorithm 3) performed poorly. Inclusion of additional information on anti‐diabetic treatment and the exclusion of women given a gestational diabetes mellitus diagnosis code in the index pregnancy increased the PPV but decreased the completeness across all algorithms. Algorithms 1 and 2 showed similar patterns in terms of number of identified cases over the years, indicating that both algorithms were robust against potential changes over time (e.g., in registries and demographics).

### 4.1 Other studies

Although previous studies have developed algorithms to identify women with type 1 diabetes in pregnancy, to the best of our knowledge, this is the first study to evaluate the validity of these algorithms using Danish registries and to confirm that the algorithms can be used on a clinical cohort. Two Danish studies from 2007 aimed to validate a national algorithm to identify any type of diabetes but not related to pregnancy and found that the national registries best identified pharmacologically treated individuals, which is the case for all individuals with type 1 diabetes.^3^, ^5^ A Norwegian study validated diabetes, asthma and epilepsy among pregnant women in Norway, using issued prescriptions as gold standard against diagnosis codes and reported a sensitivity of 90% and a PPV of 56% for type 1 diabetes, concluding that the registries best identified type 1 diabetes.^12^, ^13^

We found a 0.24% prevalence of type 1 diabetes among women giving live birth, which is in line with previous studies using similar methods. A previous Danish study reported a 0.5% prevalence between 1996 and 2016, a Scottish study reported a prevalence of 0.39% between 1998 and 2013 and a French study reported a prevalence of 0.16% in 2012.^14^, ^15^, ^16^ Three Swedish studies using the ‘preexisting type 1 diabetes in pregnancy’ diagnosis registered before delivery in the index pregnancy (ICD10‐code DO240)—corresponding to algorithm 3 in this study—to evaluate perinatal outcomes of type 1 diabetes in pregnancy found the prevalence of type 1 diabetes in pregnancy to be 0.4% between 1997 and 2012.^17^, ^18^, ^19^ This diagnosis‐code was not validated prior to the study. Our results question whether algorithm 3 is a valid diagnosis‐code to use in a similar study in Denmark, however, validity of registries might vary between different countries.

### 4.2 Strengths/limitations

A strength of this study is the nationwide design including all live births in Denmark during a six‐year period with all algorithms validated against a clinical cohort of women with confirmed type 1 diabetes as a gold standard.^2^ Danish registries provide nationwide data with a high validity and the prospective collection minimizes the risk of selection and information bias.^9^ In general, the validity of the registries relates to how well‐defined and well‐known a condition is.^20^ Women with type 1 diabetes are unlikely to remain undiagnosed and are thus unlikely to be misclassified as non‐type 1 diabetes‐individuals. On the contrary, some non‐type 1 diabetes‐individuals (e.g. mothers with gestational diabetes mellitus or type 2 diabetes) may be misclassified as having type 1 diabetes. The algorithms were based on clinical knowledge and were designed to identify and minimize this potential bias. In main algorithm 1, we included all women ever given any diabetes diagnosis code before 30 years of age, because type 1 diabetes most often is characterized by an early life debut, whereas type 2 diabetes is often diagnosed later in life. Main algorithms 2 and 3 were based on specific type 1 diabetes diagnosis codes. In later years, we have seen an increase in all types of diabetes in the general population, also among the young.^1^ New test methods have improved the correct diagnosis of type of diabetes and improved diagnostic testing and centralization of the treatment of type 1 diabetes have probably improved the validity of the registries as well as algorithm 3 in this study. On the contrary, an increase in BMI in the population as well as gestational diabetes and type 2 diabetes, and thus an increased risk of misclassification, will decrease the validity of the registries and the validity of algorithm 1 in this study. It is a limitation to the study that the validation cohort was from 1994 to 1999. A repeated validation of the registries including a more recent cohort as gold standard would have been favourable to evaluate whether the algorithms developed in this study still hold same high completeness and PPV, especially, as the prevalence of type 2 diabetes among pregnant women has increased significantly since the 1990s.^15^ Unfortunately, a more recent cohort was not available for validation.

A registry‐based case identification of type 1 diabetes in women giving live birth with both PPV and completeness above 80% is generally considered to be high, which this study showed for many sub‐algorithms. As PPV relies on the prevalence of a disease, it is difficult to achieve a very high PPV for a rare disease as type 1 diabetes, even with a completeness above 90%.^21^, ^22^ It would have been ideal to have an algorithm that both maximize PPV and completeness, but typically there is a trade‐off between these two and the interpretation and balancing will rely on the aim and purpose of a given study. In studies on prevalence and incidence, a high completeness is desirable but may include many false positives and yield a lower PPV. In comparative studies or studies that support clinical management one should prioritize having primarily true cases included (a high PPV) to minimize the risk of bias both away from and towards the null hypothesis (type 1 and type 2 errors). However, a very restrictive definition could miss many cases which could impact generalizability. The choice of algorithm used in future studies will depend on the aim and scope, as inclusion of additional information on anti‐diabetic treatment and the exclusion of women with a gestational diabetes diagnosis code in the index pregnancy increased the PPV but decreased the completeness across all algorithms and sub‐algorithms. In general, this study found the algorithms reliable for evaluating outcomes in comparative outcome studies. The algorithms may also be suitable for monitoring prevalence and incidence when considering that PPV and completeness are around 80%–90%, yet not perfect.

It is a limitation to the study that the DDBR cohort used as gold standard in the present study was only 75%–93% complete.^6^ However, during the 1990's, treatment and care for pregnant women was not yet centralized and therefore the responsibility for the data collection was managed by 8 obstetric departments introducing risk of sub‐optimal reporting to registries. In a study on the DDBR, the reported cases were compared with non‐reported cases and the two groups were found to be comparable regarding background information.^2^ The distribution of the missing cases in the DDBR cohort impacted the calculation of PPV and completeness. When correcting for the missing cases in the quantitative bias analysis, we found that we likely underestimated PPV and overestimated completeness. However, both PPV and completeness stayed above 80% after the correction for most algorithms, indicating that the missing cases did not have major effect on the algorithms. In a real world setting, the algorithms would still hold a high accuracy, even though they were tested against an incomplete gold standard.

The present study only included live births and some patients having stillbirths will be missing from the cohort. This, however, will only affect studies using the algorithm to study still‐birth rates. The DDBR cohort found a stillbirth rate of 2.1% corresponding to 21 cases during 1994 to 1999.^2^

We did not include specificity and negative predictive value in this study, as the low prevalence of type 1 diabetes in the population will cause the specificity and negative predictive value to always be close to 100%.

## 5 CONCLUSION

The present study shows that algorithms can reliably identify type 1 diabetes in pregnancy in health registries, with a PPV above 80% and completeness around 90%. The algorithms and registries are well‐suited for future comparative outcome studies and may also be suitable for monitoring prevalence and incidence when considering that the PPV and completeness are not perfect.

## AUTHOR CONTRIBUTIONS

**Tina Wullum Gundersen:** Conceptualization (equal); data curation (equal); formal analysis (lead); investigation (equal); methodology (equal); project administration (equal); software (equal); writing – original draft (equal); writing – review and editing (lead). **Andreas Ebbehoj:** Conceptualization (equal); data curation (equal); formal analysis (equal); methodology (equal); writing – review and editing (equal). **Sine Knorr:** Conceptualization (equal); methodology (equal); writing – review and editing (equal). **Dorthe Møller Jensen:** Conceptualization (equal); data curation (equal); methodology (equal); writing – review and editing (equal). **Peter Damm:** Conceptualization (equal); methodology (equal); writing – review and editing (equal). **Ellen Christine Leth Loekkegaard:** Conceptualization (equal); methodology (equal); writing – review and editing (equal). **Elisabeth Mathiesen:** Conceptualization (equal); methodology (equal); writing – review and editing (equal). **Tine D Clausen:** Conceptualization (equal); data curation (equal); formal analysis (equal); investigation (equal); methodology (equal); supervision (lead); writing – review and editing (equal).

## FUNDING INFORMATION

This research did not receive any specific grant from funding agencies in the public, commercial or not‐for‐profit sectors.

## CONFLICT OF INTERESTS

TWG, AE, SK, DMJ, ELL and RWT, declare that there is no conflict of interest associated with their contribution to this manuscript. PD and ERM are participating in multi‐centre and multi‐national clinical studies on the use of insulin in pregnant women with pre‐existing diabetes in collaboration with Novo Nordisk, no personal honorarium is involved. TDC, PD and EM are participating in clinical studies of insulin in pregnant women with pre‐existing diabetes in collaboration with Novo Nordisk based at Rigshospitalet, no personal honorarium is involved.

## ETHICS STATEMENT

The Danish Data Protection Agency approved the study (journal number NOH‐2016‐004).

## CODE AVAILABILITY

Code to reproduce the quantitative bias analysis performed in Appendix S1 is publicly available (https://github.com/andreasebbehoj/2021‐T1DM‐in‐Pregnancy‐Algorithm‐QBA).

## Supporting information


**Appendix S1:** Supporting information

## Data Availability

This study was conducted using official data from Danish registries. Accesses to the Danish national registries are provided by Statistics Denmark. Data can be accessed for research by applying for specific dataset‐extraction. No additional unpublished data from the study are publicly available.
