# Supplementary material for: Validation of Danish registry‐cases of type 1 diabetes in women giving live birth using a clinical cohort as gold standard
Source: Endocrinol Diabetes Metab. 2022 Nov 22;6(1):e374. doi: 10.1002/edm2.374 (PMC9836239; doi:10.1002/edm2.374)
Supplement: Supplementary file 1 — Appendix S1: Supporting information [file EDM2-6-e374-s001.docx]

## Appendix 1

### Limitations in using DDBR as gold standard

In the main analysis in the manuscript, we used the Danish Diabetes Birth Register (DDBR) cohort as a gold standard for validating our algorithms. In brief, the original DDBR database was prospectively collected at all obstetric departments in Denmark from 1992 to 1999. In total, 796 women with type 1 diabetes (giving 984 live births) were included in DDBR cohort within the period of the present study (1994 to 1999). Nationwide, 310,583 mothers had 407,157 live births during the study period.

All women in the DDBR were correctly diagnosed with type 1 diabetes. However, the DDBR did not include all cases of type 1 diabetes in women giving live birth. Through reviewing medical records, the authors found that the obstetric departments had only reported between 75 to 93% of all true cases of type 1 diabetes in women giving live birth from 1992-1999 ^6^. Thus, 7-25% of true cases are likely missing in the DDBR cohort used as gold standard and the true number of type 1 diabetes in women giving live birth will be between 984/0.93=1,058 and 984/0.75=1,312. Consequently, 74 to 328 cases of type 1 diabetes in women giving live birth do not appear in the DDBR cohort.

*Supplementary table 1* Distribution of cases of type 1 diabetes in women giving live birth in Denmark when assuming the DDBR cohort to be 75-93% complete

|  | All true cases of type 1 diabetes in women giving live birth in Denmark | All cases of non-type 1 diabetes in women giving live birth in Denmark | Total |
| --- | --- | --- | --- |
| Included in DDBR cohort | 984 | 0 | 984 |
| Not included in DDBR cohort | 74 to 328 | 406,099 to 405,845 | 406,173 |
| Total | 1,058 to 1,312 | 406,099 to 405,845 | 407,157 |

The missing cases in the DDBR cohort were most likely due to treatment and care of type 1 diabetes in pregnancy not yet being centralized in the 1990’s and therefore the responsibility for the data collection was spread to 8 obstetric departments. The cases of type 1 diabetes in women giving live birth in the DDBR were found to be comparable to non-reported cases regarding background information (Jensen et al., 2004).

In the following sections we used standard quantitative bias analysis methods to estimate how this misclassification bias could potentially have impacted each algorithm’s PPV and completeness ^23^.

### Efficacy of algorithms and quantitative bias analysis

In our main analysis in the manuscript, we assume that all women with type 1 diabetes giving live birth are included in the DDBR cohort, even though between 74 and 328 cases are likely missing. When calculating the efficacy of each algorithm, the missing cases will either be found among the false positives or the true negatives.

|  | Enrolled in DDBR cohort | Not enrolled in DDBR cohort | Total |
| --- | --- | --- | --- |
| Algorithm positive | True positives | False positives |  |
| Algorithm negative | False negatives | True negatives |  |
| Total | 984 | 406,173^#^ | 407,157 |

*# Including an unknown number of cases of type 1 diabetes in women giving live birth, likely between 74 and 328, as described above.*

How the missing cases of type 1 diabetes in women giving live birth affect the PPV and completeness of our algorithms depends on the number of missing cases and the distribution between false positive and true negative.

For our quantitative bias analysis, we made the following assumptions:

1. The DDBR cohort included either 75 or 93% of all true cases of type 1 diabetes in women giving live birth, corresponding to 328 and 74 missing cases, respectively.
2. We calculated a best estimate, assuming the average, uncorrected PPV for all algorithms (76%) also applies for cases of type 1 diabetes in women giving live birth missing from the DDBR cohort (meaning that 76% of missing cases of type 1 diabetes in women giving live birth were found in false positives and will be moved to true positives and 27% were true negatives and will be moved to false negatives).
3. We calculated the range from best- to worst-case scenario. In the best-case scenario, we assumed that our algorithms identified 100% of the missing cases (meaning all missing cases of type 1 diabetes in women giving live birth were found in false positives and will be moved to true positives). In the worst-case scenario, we assumed that our algorithms identified 0% of the missing cases (meaning all cases of type 1 diabetes in women giving live birth were found in true negatives and will be moved to false negatives).

We found it unlikely that any algorithm could have no false positives (i.e. achieve a PPV of 100%). Therefore, we capped the maximum number of cases that could be moved from false positive to true positive to 76% of the uncorrected false positive for each algorithm.

An example of the calculations is provided below. All calculations were conducted in Stata version 16.1 (StataCorp LLC, College Station, Texas, USA). The code to reproduce the analysis is publicly available (<https://github.com/andreasebbehoj/2021-T1DM-in-Pregnancy-Algorithm-QBA>).

### Example on how to calculate corrected PPV and completeness

In the figure below, we show an example of how to calculate uncorrected and corrected PPV and completeness for a single algorithm (main algorithm 1) when assuming that DDBR was 75% complete (figure 1). The three corrected calculations show estimates when 0%, 76% or 100% were identified by the algorithm.

*Supplementary figure 1* Calculations of corrected PPV and completeness when assuming DDBR included 75% of all true cases of T1DM in pregnancy (missing 328 cases).


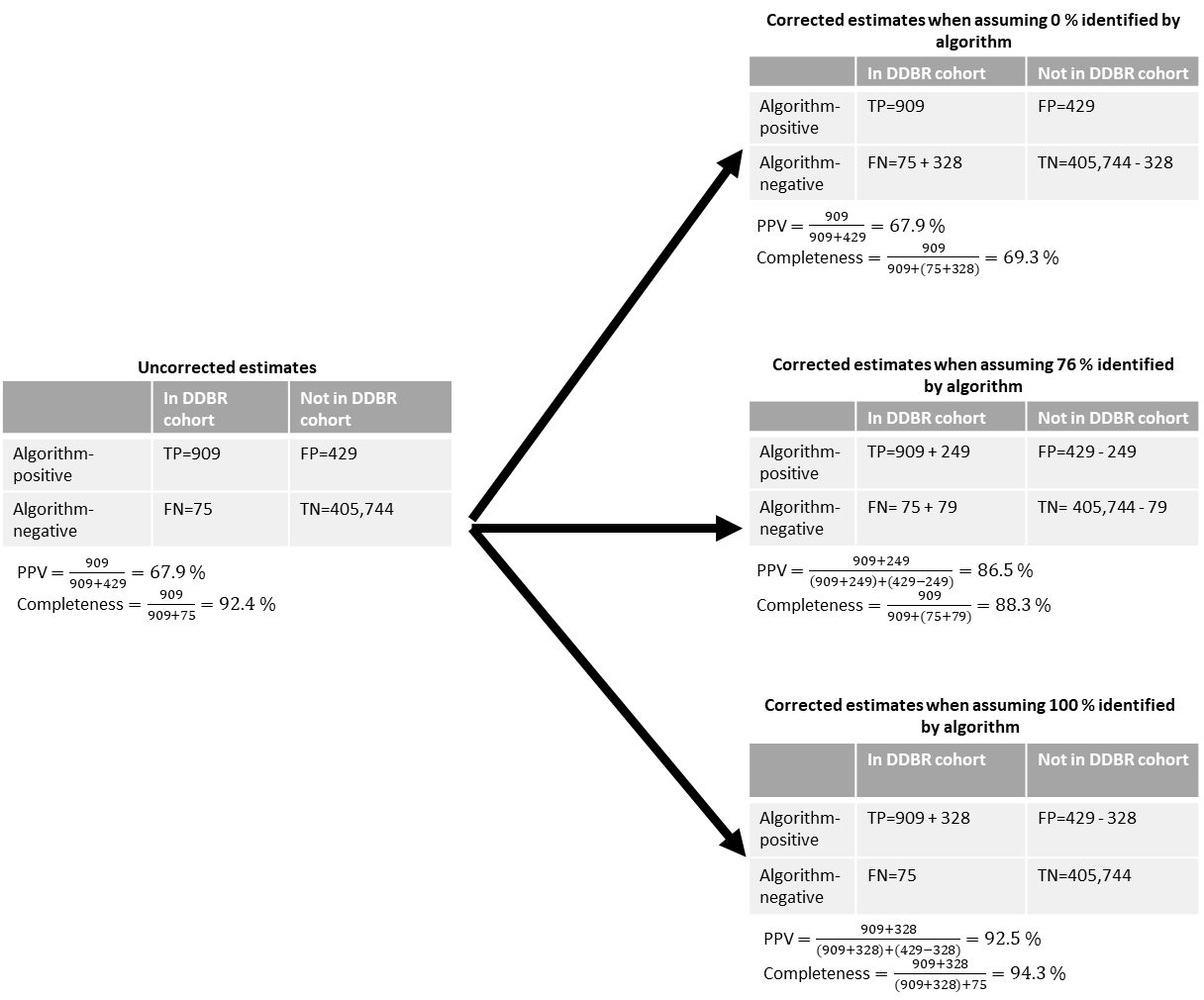


Abbreviations: TP= true positive, FP = false positive, FN = false negative, TN = true negative, PPV = positive predictive value

In this example, the best estimates for PPV and completeness for main algorithm 1, when assuming the DDBR cohort to be 75 % complete, were 86.5 % (uncorrected 69.7 %) and 88.3 % (uncorrected 92.4 %), respectively. We calculated a corrected PPV in a range from 67.9 % and 92.5 % and a corrected completeness in a range from 69.3 to 94.3 %. The true PPV and completeness, when assuming that DDBR is 75 % correct, is within this calculated range.

### Results

Uncorrected and corrected results for all algorithms are shown in Supplementary table 2 below.

*Supplementary table 2* Uncorrected and corrected PPV and completeness of all algorithms with best estimates and ranges. Best estimates are calculated assuming that the average, uncorrected PPV (76%) for all algorithms also applies for all true cases of type 1 diabetes in women giving live birth missing from the DDBR cohort. Ranges for corrected estimates indicates results when assuming the uncorrected PPV of the missing cases are 0% and 100% respectively.

|  | *Uncorrected* | | *Corrected, DDBR 93% complete* | | *Corrected, DDBR 75% complete* | |
| --- | --- | --- | --- | --- | --- | --- |
| *Algorithms* | *PPV, %* | *Completeness,*  *%* | *PPV:*  *best estimate*  *% (range)* | *Completeness,*  *best estimate*  *% (range)* | *PPV,*  *best estimate*  *% (range)* | *Completeness,*  *best estimate*  *% (range)* |
| Algorithm 1: any diabetes diagnosis registered in NPR before delivery in the index pregnancy and registered before maternal age of 30 | 67.9 | 92.4 | 72.1 (67.9-73.5) | 91.2 (85.9-92.9) | 86.5 (67.9-92.3) | 88.3 (69.3-94.1) |
| 1. redeemed at least one prescription of insulin | 76.7 | 92.4 | 81.4 (76.7-83.0) | 91.2 (85.9-92.9) | 94.4 (76.7-94.4) | 85.3 (69.3-85.3) |
| 1. redeemed a prescription of insulin before oral antidiabetics | 78.7 | 91.2 | 83.6 (78.7-85.2) | 90.1 (84.8-91.8) | 94.9 (78.7-94.9) | 82.5 (68.4-82.5) |
| 1. redeemed a prescription of insulin and never redeemed a prescription of oral antidiabetics | 81.8 | 84.6 | 87.3 (81.8-89.1) | 83.9 (78.6-85.6) | 95.7 (81.8-95.7) | 74.2 (63.4-74.2) |
| 1. never redeemed a prescription of oral antidiabetics | 67.8 | 92.4 | 72.0 (67.8-73.3) | 91.2 (85.9-92.9) | 86.4 (67.8-92.2) | 88.3 (69.3-94.3) |
| 1. never given a diagnosis of GDM in the index pregnancy | 70.0 | 88.5 | 74.5 (70.0-75.9) | 87.6 (82.3-89.3) | 90.0 (70.0-92.8) | 85.4 (66.4-88.0) |
| 1. never redeemed a prescription of oral antidiabetics and who were never given a diagnosis of GDM in the index pregnancy | 73.4 | 83.9 | 78.3 (73.4-79.9) | 83.4 (78.1-85.1) | 93.6 (73.4-93.6) | 80.3 (63.0-80.3) |
| Algorithm 2: a specific “type 1 diabetes” diagnosis registered in NPR before delivery regardless of maternal age in the index pregnancy | 77.4 | 92.4 | 82.1 (77.4-83.7) | 91.2 (85.9-92.9) | 94.6 (77.4-94.6) | 84.7 (69.3-84.7) |
| 1. redeemed at least one prescription of insulin | 79.0 | 92.4 | 83.8 (79.0-85.4) | 91.2 (85.9-92.9) | 95.0 (79.0-95.0) | 83.3 (69.3-83.3) |
| 1. redeemed a prescription of insulin before oral antidiabetics | 75.1 | 91.5 | 79.8 (75.1-81.3) | 90.4 (85.1-92.1) | 94.0 (75.1-94.0) | 85.8 (68.6-85.8) |
| 1. redeemed a prescription of insulin and never redeemed a prescription of oral antidiabetics | 87.6 | 84.3 | 93.5 (87.6-95.4) | 83.7 (78.4-85.4) | 97.0 (87.6-97.0) | 70. 1 (63.3-70.1) |
| 1. never redeemed a prescription of oral antidiabetics | 79.9 | 84.3 | 85.3 (79.9-87.0) | 83.7 (78.4-85.4) | 95.2 (79.9-95.2) | 75.4 (63.3-75.4) |
| 1. never given a diagnosis of GDM in the index pregnancy | 80.4 | 87.2 | 85.7 (80.4-87.3) | 86.4 (81.1-88.1) | 95.3 (80.4-95.3) | 77.5 (65.4-77.5) |
| 1. never redeemed a prescription of oral antidiabetics and who were never given a diagnosis of GDM in the index pregnancy | 81.6 | 80.0 | 87.4 (81.6-89.3) | 79.7 (74.4-81.4) | 95.6 (81.6-95.6) | 70.3 (60.0-70.3) |
| Algorithm 3 a “preexisting type 1 diabetes in pregnancy” diagnosis registered in NPR before delivery regardless of maternal age in the index pregnancy | 65.7 | 84.5 | 70.1 (65.7-71.5) | 83.8 (78.5-85.5) | 85.4 (65.7-91.6) | 82.3 (63.3-88.3) |
| 1. redeemed at least one prescription of insulin | 71.6 | 84.5 | 76.4 (71.6-78.0) | 83.8 (78.5-85.5) | 93.0 (71.6-93.2) | 82.3 (63.3-82.5) |
| 1. redeemed a prescription of insulin before oral antidiabetics | 74.3 | 83.3 | 79.3 (74.3-81.0) | 82.8 (77.5-84.5) | 93.8 (74.3-93.8) | 79.0 (62.5-79.0) |
| 1. redeemed a prescription of insulin and never redeemed a prescription of oral antidiabetics | 79.7 | 76.3 | 85.7 (79.7-87.6) | 76.3 (71.0-78.0) | 95.1 (79.7-95.1) | 68.3 (57.2-68.3) |
| 1. never redeemed a prescription of oral antidiabetics | 74.4 | 76.3 | 80.0 (74.4-81.8) | 76.3 (71.0-78.0) | 93.9 (74.4-93.9) | 72.2 (57.2-72.2) |
| 1. never given a diagnosis of GDM in the index pregnancy | 73.9 | 81.1 | 79.1 (73.9-80.7) | 80.7 (75.4-82.4) | 93.7 (73.9-93.7) | 77.1 (60.8-77.1) |
| 1. never redeemed a prescription of oral antidiabetics and who were never given a diagnosis of GDM in the index pregnancy | 79.3 | 73.6 | 85.4 (79.3-87.4) | 73.7 (68.4-75.4) | 95.1 (79.3-95.1) | 66.2 (55.2-66.2) |

Abbreviations: PPV: positive predictive value. PPV defined as true positive divided by all algorithm positive (including false positives). Completeness defined as true positive divided by all true cases of type 1 diabetes in women giving live birth (including false positives).
